# Validity and reliability of short‐term heart‐rate variability from disposable electrocardiography leads

**DOI:** 10.1002/hsr2.984

**Published:** 2022-12-08

**Authors:** Nduka C. Okwose, Sophie L. Russell, Mushidur Rahman, Charles J. Steward, Amy E. Harwood, Gordon McGregor, Srdjan Ninkovic, Helen Maddock, Prithwish Banerjee, Djordje G. Jakovljevic

**Affiliations:** ^1^ Cardiovascular and Lifestyle Medicine Research Theme, Faculty Research Centre (CSELS), Institute for Health and Wellbeing Coventry University Coventry UK; ^2^ Department of Cardiology University Hospitals Coventry and Warwickshire NHS Trust Coventry UK; ^3^ Department of Surgery, Clinical Centre, Faculty of Medical Sciences University of Kragujevac Kragujevac Serbia

**Keywords:** disposable leads, ECG, heart rate variability, reproducibility, validity

## Abstract

**Background and Aims:**

Single‐use electrocardiography (ECG) leads have been developed to reduce healthcare‐associated infection. This study compared the validity and reliability of short‐term heart rate variability (HRV) obtained from single‐use disposable ECG leads.

**Methods:**

Thirty healthy subjects (33 ± 10 years; 9 females) underwent 5‐min resting HRV assessments using disposable (single use) ECG cable and wire system (Kendall DL™ Cardinal Health) and a standard, reusable ECG leads (CardioExpress, Spacelabs Healthcare).

**Results:**

Intraclass correlation coefficient (ICC) with 95% confidence interval (CI) between disposable and reusable ECG leads was for the time domain [R‐R interval (ms); 0.99 (0.91, 1.00)], the root mean square of successive normal R‐R interval differences (RMSSD) (ms); 0.91 (0.76, 0.96), the SD of normal‐to‐normal R‐R intervals (SDNN) (ms); 0.91 (0.68, 0.97) and frequency domain [low‐frequency (LF) normalized units (nu); 0.90 (0.79, 0.95), high frequency (HF) nu; 0.91 (0.80, 0.96), LF power (ms^2^); 0.89 (0.62, 0.96), HF power (ms^2^); 0.90 (0.72, 0.96)] variables. The mean difference and upper and lower limits of agreement between disposable and reusable leads for time‐ and frequency‐domain variables were acceptable. Analysis of repeated measures using disposable leads demonstrated excellent reproducibility (ICC 95% CI) for R‐R interval (ms); 0.93 (0.85, 0.97), RMSSD (ms); 0.93 (0.85, 0.97), SDNN (ms); 0.88 (0.75, 0.95), LF power (ms^2^); 0.87 (0.72, 0.94), and HF power (ms^2^); 0.88 (0.73, 0.94) with coefficient of variation ranging from 2.2% to 5% (*p* > 0.37 for all variables).

**Conclusion:**

Single‐use Kendall DL™ ECG leads demonstrate a valid and reproducible tool for the assessment of HRV.

## 1 INTRODUCTION

Heart‐rate variability (HRV), which represents changes in the time intervals between consecutive heartbeats, is a recognized physiological indicator of the autonomic nervous system (ANS) activity.^1^ It reflects neuro‐cardiac interactions and dynamic nonlinear ANS processes which operate on different time scales to maintain neuro‐cardiac homeostasis.^2^

The evaluation of HRV has gained significant interest as a simple, noninvasive measure sensitive to physiological and psychological variations.^3^ It provides an insight into the human heart's capacity to adapt to complex physiological, pathophysiological, or environmental challenges.^3^ Hence, HRV has been evaluated and reported to predict mortality in the general population,^4^ improve risk stratification in cardiovascular disease,^5^, ^6^, ^7^ diabetes,^8^ sleep deficiency,^9^ cancer,^10^ and mental health disorder.^11^, ^12^ In athletes, it has been proposed that HRV measurement can guide exercise training programs, adaptation, and recovery.^13^, ^14^

Depending on the methodology and purpose, HRV is commonly monitored briefly (ultrashort‐term, <5 min), short‐term (~5 min), or during a 24‐h period and evaluated using time‐domain, frequency‐domain, and nonlinear measurements.^2^ While the heart's response to a wider range of environmental stimuli and workloads is sometimes assessed during short‐term monitoring, long‐term monitoring is representative of processes with slower fluctuations (e.g., circadian rhythm) and is not exchangeable with short‐term values.^2^

Time domain measures of HRV quantify variability in beat‐to‐beat interval measurements. For example, the standard deviation of normal‐to‐normal R‐R intervals (SDNN); a representation of beat‐to‐beat intervals without artefacts, provides estimates of overall HRV, and the root mean square of successive beat‐to‐beat interval differences (RMSSD), gives an indication of variations in parasympathetic activity.^13^ These two, are the only time‐domain measures recommended for short‐term HRV analysis.^15^ Frequency‐domain measures evaluate the distribution of absolute or relative power into frequency bands, that is, ultralow‐frequency (ULF), very‐low‐frequency (VLF), low‐frequency (LF), and high‐frequency (HF) bands.^15^ The LF band, mediated by baroreflex mechanisms,^16^ is associated with sympathetic and parasympathetic modulations. The HF band reflects the respiratory sinus rhythm from centrally mediated cardiac vagal control.^13^

Although microbial contamination of the fomite electrocardiographic telemetry systems is significantly reduced after cleaning,^17^ ECG wires cannot be completely disinfected 100% of the time due to the frequency of usage between patients. Thus, they may contribute to the growth of resistant bacteria in clinical settings.^18^ Single‐use, disposable electrocardiography (ECG) leads have been developed to serve as a means of reducing the transfer of disease‐causing agents to a new human host. Their validity and reliability in assessing heart rate variability (HRV) have not yet been reported. This study aimed to assess the validity and reliability of short‐term HRV measures obtained from the novel single‐use ECG leads. The present study tested the following two logical alternative hypotheses: (i) HRV time‐ and frequency‐domain measures obtained by a disposable ECG lead will be significantly correlated and in agreement with those obtained by the standard, reusable ECG leads and (ii) disposable ECG leads will demonstrate acceptable reproducibility.

## 2 METHODS

### 2.1 Study design and procedure

This was a single‐center, prospective, observational validity, and reliability study approved by the Coventry University Institute of Health and Wellbeing Research Ethics Committee (P109193). The study protocol and procedures were in accordance with the declaration of Helsinki, and participants provided written informed consent before taking part in the study. To ensure a high level of consistency in the physiological status of participants, individuals without a history of cardiovascular, respiratory, or other chronic or acute illnesses participated in the study. Participants attended the Cardiovascular Research Laboratory, for two visits lasting approximately 1.5 h each. All participants were asked to refrain from alcohol and caffeine on the study day and to avoid vigorous exercise 24 h before the study visit as these could alter HRV. While supine, participants’ blood pressure and heart rate were measured, and participants’ skin was prepared for the attachment of the ECG electrodes. The electrodes were placed on the participant's thorax by trained technicians using the standard 12‐lead placement^19^ and then connected to the CardioExpress transducer. HRV measurements were taken from the lead II under light‐controlled, thermo‐neutral, and calm conditions. HRV was assessed under normal breathing conditions simultaneously for 5 min each at rest using a single‐use disposable ECG cable and lead wire system (Kendall DL™, Cardinal Health) and reusable ECG leads (CardioExpress SL18A, Spacelabs Healthcare). The CardioExpress system with reusable leads was chosen to provide criterion measures of HRV as an established technology for cardiac rhythm assessment in clinical settings. The sampling frequency was automatically set at 100 Hz according to manufacturer recommendations.

Measurements were repeated 1 week apart using the same approach, to assess the reliability of disposable leads in assessing HRV. Signal processing and generation of R‐R interval variability data were made automatically by the CardioExpress software. HRV measurements included the following variables: heart rate, R‐R interval, SDNN, RMSSD, HF power (in absolute and normalized units), and LF power (in absolute and normalized units).

### 2.2 Statistical analysis

Descriptive statistics were calculated for all variables. Data are presented as mean ± SD. Normal distribution was assessed via visual inspection of frequency histogram and with either a Kolmogorov–Smirnov or Shapiro–Wilk test. Heart rate, R‐R intervals, LF power, and HF power in normalized units were analyzed using raw data, whereas RMSSD, SDNN, LF power, and HF power were analyzed after log transformation due to non‐normal distribution. The agreement between disposable and reusable leads was performed using Bland–Altman analysis (i.e., mean difference [upper and lower limits of agreement]), intraclass correlation coefficient (ICC), and Pearson's coefficient of correlation. An ICC and Pearson's coefficient of correlation value above 0.9 was considered excellent correlation, while value ≥ 0.7 < 0.9 was considered very good. Test–retest reliability of the disposable leads was performed using ICC and coefficient of variation (CoV). CoV (%) was used to measure the degree of dispersion around the mean of repeated measurements and was calculated as the standard deviation of repeated measurements expressed as a percentage of the mean. Data were analyzed using SPSS (Version 27).

## 3 RESULTS

Thirty healthy participants were recruited for the study. The physical characteristics of participants are presented in Table 1. The total number of beats detected after the automatic removal of artefacts by CardioExpress software was 9356 for reusable leads and 9119 for disposable leads (*p* > 0.05).

**Table 1:** Demographic and physical characteristics of the study participants

Parameter	Mean ± SD
Age (years)	33 ± 10
Male/female	21/9
Weight (kg)	78.3 ± 14.0
Height (cm)	174.9 ± 8.5
Body mass index (kg/m^2^)	25.5 ± 4.0
Systolic blood pressure (mmHg)	125 ± 12
Diastolic blood pressure (mmHg)	79 ± 8

**Figure 1:**
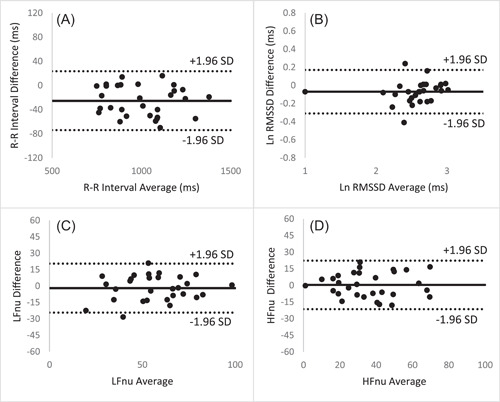
Bland–Altman plots with mean difference (solid line) and 95% limits of agreement (dotted lines) demonstrating the validity of heart rate variability using disposable electrocardiography leads compared to reusable electrocardiography leads. Variables include (A) R‐R Interval, (B) log‐transformed root mean square of successive normal R‐R intervals (RMSSD), (C) low‐frequency power in normalized units, and (D) high‐frequency power in normalized units.

ICC with 95% confidence interval (CI) between disposable and reusable ECG leads was excellent for the time domain [R‐R interval (ms); 0.99 (0.91, 1.00), RMSSD (ms); 0.91 (0.76, 0.96), SDNN (ms); 0.91 (0.68, 0.97)] and very good to excellent for frequency domain [LF nu; 0.90 (0.79, 0.95), HF nu; 0.91 (0.80, 0.96), LF power (ms^2^); 0.89 (0.62, 0.96), HF power (ms^2^); 0.90 (0.72, 0.96)] variables (Table 2). Agreement between both leads showed small mean differences and narrow limits of agreement for time domain [R‐R interval (ms); −25.5 (−74.4, 23.5), RMSSD (ms); −0.07 (−0.31, 0.17), SDNN (ms); −0.07 (−0.25, 0.12)] and frequency domain [LF nu; −1.9 (−24.3, 20.4), HF nu; 0.5 (−21.4, 22.3), LF power (ms^2^); −0.18 (−0.68, 0.32), HF power (ms^2^); −0.16 (−0.68, 0.37)] variables (Table 2). Bland–Altman plots for R‐R intervals, RMSSD, LF nu, and HF nu are presented in Figure 1.

**Table 2:** Comparison of time and frequency domain variables between reusable and disposable leads (*n* = 30)

	Mean ± SD		% Difference	Mean difference (LOA)	ICC (95% CI)
Parameter	Reusable leads	Disposable leads			
Raw data					
HR (bpm)	61.9 ± 11.2	60.3 ± 10.7	2.6	1.6 (−2.1, 5.3)	1.00 (0.92, 1.00)
R‐R Interval (ms)	991 ± 176	1016 ± 179	2.6	−25.5 (−74.4, 23.5)	0.99 (0.91, 1.00)
LF nu	56.0 ± 19.4	57.9 ± 18.2	3.4	−1.9 (−24.3, 20.4)	0.90 (0.79, 0.95)
HF nu	36.5 ± 18.8	36.0 ± 18.9	1.4	0.5 (−21.4, 22.3)	0.91 (0.80, 0.96)
Log‐transformed data					
RMSSD (ms)	2.57 ± 0.26	2.64 ± 0.22	2.7	−0.07 (−0.31, 0.17)	0.91 (0.76, 0.96)
SDNN (ms)	2.67 ± 0.21	2.74 ± 0.18	2.6	−0.07 (−0.25, 0.12)	0.91 (0.68, 0.97)
LF power (ms^2^)	3.68 ± 0.53	3.86 ± 0.39	4.9	−0.18 (−0.68, 0.32)	0.89 (0.62, 0.96)
HF power (ms^2^)	3.39 ± 0.52	3.55 ± 0.46	4.7	−0.16 (−0.68, 0.37)	0.90 (0.72, 0.96)

Abbreviations: bpm, beats per minute; CI, confidence interval; HF, high‐frequency power; HR, heart rate; ICC, intraclass coefficient of correlation; LF, low‐frequency power; LOA, limits of agreement; ms, milliseconds; ms^2^, absolute units; nu, normalized units; RMSSD, root mean square of successive differences, square root of the mean sum of squared differences between the duration of all normal successive R‐R‐intervals; R‐R, count number of mean time between R waves; SDNN, standard deviation of the duration of all normal R‐R‐intervals.

**Figure 2:**
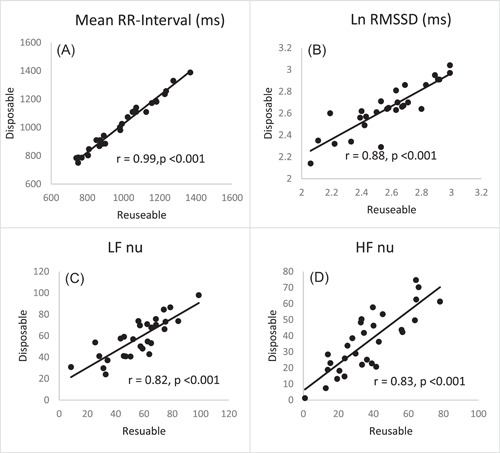
Relationship between reusable and disposable leads: (A) R‐R Interval, (B) log‐transformed root mean square of successive normal R‐R intervals (RMSSD), (C) low‐frequency power in normalized units, and (D) high‐frequency power in normalized units.

Repeated measures of disposable leads demonstrated excellent reproducibility (ICC 95% CI) for time‐domain variables [R‐R interval (ms); 0.93 (0.85, 0.97), RMSSD (ms); 0.93 (0.85, 0.97), SDNN (ms); 0.88 (0.75, 0.95)] with CoV <5%. Frequency domain variables also showed very good to excellent reproducibility [LF nu; 0.77 (0.52, 0.89), HF nu; 0.74 (0.44, 0.86), LF power (ms^2^); 0.87 (0.72, 0.94), and HF power (ms^2^); 0.88 (0.73, 0.94)] (Table 3) although CoV was higher than the acceptable 10% limit for normalized units of LF and HF power (Table 3). Relationships between repeated measures of R‐R intervals, RMSSD, LF nu, and HF nu are presented in Figure 2.

**Table 3:** Reproducibility of time and frequency domains variables using disposable leads (*n* = 30)

	Mean (SD)		ICC (95% CI)	CoV (%)
Parameter	Visit 1	Visit 2		
Raw data				
HR (bpm)	60.3 ± 10.7	60.7 ± 11.3	0.95 (0.89, 0.97)	4.8
R‐R Interval (ms)	1016 ± 179	1012 ± 184	0.93 (0.85, 0.97)	4.8
LF nu	57.9 ± 18.2	55.4 ± 21.7	0.77 (0.52, 0.89)	19
HF nu	36.0 ± 18.9	35.7 ± 20.9	0.74 (0.44, 0.86)	30
Log‐transformed data				
RMSSD (ms)	2.65 ± 0.22	2.63 ± 0.25	0.93 (0.85, 0.97)	2.8
SDNN (ms)	2.75 ± 0.18	2.74 ± 0.16	0.88 (0.75, 0.95)	2.2
LF power (ms^2^)	3.86 ± 0.39	3.82 ± 0.40	0.87 (0.72, 0.94)	3.8
HF power (ms^2^)	3.55 ± 0.46	3.52 ± 0.54	0.88 (0.73, 0.94)	5.1

Abbreviations: bpm, beats per minute; CI, confidence interval; CoV, coefficient of variation; HF, high‐frequency power; HR, heart rate; ICC, intraclass coefficient of correlation; LF, low‐frequency power; ms, milliseconds; ms^2^, absolute units; nu, normalized units; RMSSD, root mean square of successive differences, square root of the mean sum of squared differences between the duration of all normal successive R‐R‐intervals; RR, count number of mean time between R waves; SDNN, standard deviation of the duration of all normal to normal R‐R‐intervals.

## 4 DISCUSSION

This is the first study to evaluate the validity and reliability of disposable ECG leads for measuring. The main findings of the present investigation suggest: (i) Excellent agreement between disposable and reusable ECG leads in the assessment of time and frequency domain HRV measures and (ii) disposable ECG leads demonstrated an excellent degree of reliability for time domain HRV measures, and very good to excellent reliability for frequency domain HRV measures.

Over the past two decades, several investigations have scrutinized the validity and reliability of commercially available technologies for the assessment of HRV.^20^, ^21^, ^22^, ^23^, ^24^ However, these devices are mainly designed for fitness/athletic purposes and may have little use in clinical settings. This is the first study to provide evidence on the performance accuracy of the novel; disposable ECG leads to evaluate HRV, which is a highly sensitive physiological measure used in a clinical setting for risk stratification and monitoring purposes.

Evidence from available literature assessing multiple technologies suggests large discrepancies in the design and analytical methods used to analyze time and frequency domain variables leading to conflicting results and difficulty in making comparisons among studies. For example, some studies^20^, ^25^, ^26^ analyzed HRV data using independent commercially available software to interpret R‐R interval data, rather than the specific analytical tools designed, and recommended by the device hardware provider. Our study is unique as HRV data are collected using disposable and reusable ECG leads but data were analyzed using the same analytical methods. Thus, spectral decomposition of R‐R interval sequence was automatically performed by the CardioExpress software using Fast Fourier Transformation (FFT). FFT is frequently used in reliability studies^26^, ^27^, ^28^ and enables the analysis of the components of the power spectrum density to be segmented into different frequency bands for further analysis. This contrasts with autoregressive modeling, which is dependent on criterion‐defined models and algorithms. However, there is evidence to show that data from FFT and autoregressive spectral estimation methods agree well.^29^

Although there is no direct comparison with previous studies due to the novelty, all HRV variables assessed in this study fall within the normative range reported by previous studies.^30^ Our results show a similar trend for method agreement and reliability with other studies for time‐domain HRV measures derived from short‐term recordings.^20^, ^26^ It was previously recommended to use higher sampling frequencies, usually from 500 Hz, for HRV evaluations.^15^ However, such high frequency is often impractical in clinical settings due to technological limitations of certain medical devices (e.g., Holter monitors), which are compact with small battery capacities, thus limiting sampling frequencies to around 125 Hz.^31^ Such limitations have led to the down‐sampling of frequencies up to 50 Hz^32^, ^33^ further complicating results. While a previous report suggested a sampling frequency of 50 Hz was acceptable for time domain variables,^34^ others suggest a higher cut‐off frequency for valid measurements of 100 Hz for the time domain and 250 Hz for the frequency domain variables.^33^ Below 250 Hz, frequency domain variables may lose small details of R‐R intervals due to poor concordance particularly in HF elements.^33^ This could be the reason for high CoV in normalized units of low and HF power in the present study. Variations in frequency domain measures across repeated tests, despite strict control measures, have led to reservations in the utility of frequency variables in assessing treatment effects.^24^, ^35^, ^36^ While some extrinsic factors such as time and conditions of testing can be controlled, intrinsic factors like mental activity, mood, and ectopic rhythm are difficult to control and are potential sources of random error irrespective of measurement instrumentation.

The present study is not without limitations. The reliability of the disposable ECG leads was assessed by comparing the original HRV values to values obtained 1‐week later. Although our results showed excellent reliability, any inherent error may have been masked by the natural variation exhibited by participants during the second measurement.

In conclusion, the present study has shown that disposable ECG leads are a valid tool for evaluating HRV. The present study's authors agree with other studies suggesting that HRV variables should always be measured using the same device during HRV assessments.^26^, ^35^ It is also important to be aware of the algorithm used for calculating HRV variables while designing future studies or establishing criteria for reference values.

## AUTHOR CONTRIBUTIONS

**Nduka C. Okwose**: Formal analysis; investigation; supervision; writing – original draft; writing – review and editing. **Sophie L. Russell**: Formal analysis; investigation; writing – review and editing. **Mushidur Rahman**: Formal analysis; investigation. **Charles J. Steward**: Investigation. **Amy E. Harwood**: Investigation; writing – review and editing. **Gordon McGregor**: Writing – review and editing. **Srdjan Ninkovic**: Writing – review and editing. **Helen Maddock**: Writing – review and editing. **Prithwish Banerjee**: Writing – review and editing. **Djordje G. Jakovljevic**: Conceptualization; funding acquisition; investigation; methodology; project administration; writing – review and editing.

## CONFLICT OF INTEREST

The authors declare no conflict of interest.

## TRANSPARENCY STATEMENT

The lead author Djordje G. Jakovljevic affirms that this manuscript is an honest, accurate, and transparent account of the study being reported; that no important aspects of the study have been omitted; and that any discrepancies from the study as planned (and, if relevant, registered) have been explained.

## Data Availability

The corresponding author shall provide data upon reasonable request. All authors have read and approved the final version of the manuscript. Djordje G. Jakovljevic had full access to all the data in this study and took complete responsibility for the integrity of the data and the accuracy of the data analysis.
